# O Efeito da Cirurgia de Revascularização Miocárdica na Função Contrátil e Sintomas em Pacientes com Disfunção Ventricular Esquerda

**DOI:** 10.36660/abc.20240486

**Published:** 2025-04-17

**Authors:** Fernando Bassan, Roberto Esporcatte, Marcelo Goulart Correia, Octavio Drummond Guina, Guilherme de Souza Weigert, Gracielle Christine do Nascimento Oliveira

**Affiliations:** 1 Instituto Nacional de Cardiologia Rio de Janeiro RJ Brasil Instituto Nacional de Cardiologia, Rio de Janeiro, RJ – Brasil; 2 Universidade do Estado do Rio de Janeiro Rio de Janeiro RJ Brasil Universidade do Estado do Rio de Janeiro - Doenças do Tórax, Rio de Janeiro, RJ – Brasil

**Keywords:** Síndrome Coronariana Crônica, Insuficiência Cardíaca, Disfunção Ventricular Esquerda, Revascularização Miocárdica

## Abstract

**Fundamento:**

O grau de disfunção do ventrículo esquerdo (VE) é um fator de risco independente para piores desfechos em pacientes com síndrome coronariana crônica (SCC). A cirurgia de revascularização miocárdica (CRVM) é a estratégia terapêutica padrão na insuficiência cardíaca isquêmica para melhorar os sintomas e o prognóstico. No entanto, os preditores de melhora ainda são incertos.

**Objetivos:**

Avaliar o efeito da revascularização miocárdica sobre a função do VE e sintomas em pacientes com SCC e fração de ejeção do ventrículo esquerdo (FEVE) reduzida, e identificar os preditores de melhora.

**Métodos:**

Analisamos, retrospectivamente, dados e condição clínica de 136 pacientes consecutivos com FEVE <50%, submetidos à CRVM. Durante o acompanhamento ecocardiográfico, a função do VE foi reavaliada no curto (3,6 meses) e longo prazo (30,8 meses), e comparada com os dados basais.

**Resultados:**

A FEVE média no pré-operatório foi 40,9 ± 8,6% e a média do escore do índice de motilidade (WMSI) foi 1,99 ± 0,36, ambos melhorando no longo prazo para 48,1 ± 15,0% (p<0,001) e 1,75 ± 0,49 (p<0,001), respectivamente. Observamos que 55,7% dos pacientes apresentaram uma melhora na FEVE ≥10% e 58,1% no WMSI ≥10%. A análise de regressão logística revelou que a doença cerebrovascular foi a única variável preditora de melhora na FEVE. No final do acompanhamento, observamos uma redução na taxa de pacientes em classe funcional III/IV em comparação ao basal (65,4 vs. 10,3% - p<0,001).

**Conclusões:**

Pacientes com SCC e FEVE reduzida, submetidos à CRVM apresentaram melhora na função contrátil e no tamanho do VE, com resposta benéfica sobre a classe funcional.

## Introdução

A cardiomiopatia isquêmica continua sendo a principal causa de morte no mundo, e o grau de disfunção do ventrículo esquerdo (VE) é um dos marcadores prognósticos. A etiologia da insuficiência cardíaca é um importante determinante, uma vez que pacientes com isquemia miocárdica apresentam uma menor sobrevida em comparação a pacientes não-isquêmicos.^1-4^

Na insuficiência cardíaca, pacientes que tiveram melhora na fração de ejeção do ventrículo esquerdo (FEVE) apresentaram melhor prognóstico.^5^ A cirurgia de revascularização miocárdica (CRVM) é indicada para uma maior sobrevida, mas o mecanismo é incerto, uma vez que nem a presença de isquemia nem a viabilidade prediz o benefício.^6,7^

Neste estudo, nosso objetivo foi analisar o efeito da CRVM sobre a função do VE em pacientes com cardiomiopatia isquêmica e fração de ejeção reduzida.

## Métodos

### População do estudo

Este foi um estudo retrospectivo, observacional, do tipo coorte, de pacientes consecutivos e não selecionados, com cardiomiopatia isquêmica submetidos à CRVM entre 2013 e 2017 em nossa instituição. Os pacientes incluídos apresentavam (1) FEVE<50% avaliada por ecocardiograma no período pré-operatório; (2) nenhuma necessidade de intervenção valvar associada ou reconstrução do VE. Uma vez que eventos isquêmicos agudos possam ser um forte viés para a recuperação da função do VE, também excluímos pacientes com síndrome coronariana aguda dois meses antes da cirurgia.

### Manejo do paciente

A decisão sobre indicação de CRVM foi feita pela equipe multidisciplinar composta por cardiologistas clínicos e cirurgiões. O Euroscore II foi usado para avaliar o risco pré-operatório. Acidente vascular cerebral pós-operatório foi definido como um evento novo ou em progressão que persistiu por mais de 24 horas durante o período de internação. Infarto do miocárdio pós-operatório foi definido de acordo com a terceira definição universal de infarto do miocárdio.^8^ A doença cerebrovascular foi definida como estenose da carótida ≥70% unilateral ou ≥50% bilateral. Após a hospitalização, os pacientes foram acompanhados regularmente no ambulatório. Realizamos uma avaliação de todos os pacientes no final do período de acompanhamento para avaliar seus status clínico para análise neste estudo.

### Análise ecocardiográfica

Os pacientes foram submetidos ao ecocardiograma transtorácico (ETT) de repouso antes da CRVM (basal), nos primeiros seis meses após a cirurgia (curto prazo) e ao final do seguimento (longo prazo). Os parâmetros do ETT foram medidos seguindo recomendações padronizadas.^9^ O escore do índice de motilidade do ventrículo esquerdo (WMSI, do inglês *wall motion score index*) foi medido usando um modelo de 17 segmentos. O seguinte escore numérico foi aplicado a cada segmento da parede de acordo com sua função contrátil avaliada visualmente: 1=normal; 2=hipocinético; 3=acinético; 4=discinético. O WMSI foi calculado como a soma de todos os escores dividida pelo número total de segmentos.

### Análise estatística

Os dados categóricos foram descritos como frequências e porcentagens. As variáveis contínuas foram avaliadas quanto à normalidade usando o teste de Shapiro-Wilk e descritas como média e desvio padrão ou mediana e intervalo interquartil. O teste do qui-quadrado e o teste de Fisher foram usados para determinar associações estatísticas entre as variáveis de interesse. O teste ANOVA de medidas repetidas e o teste de Kruskal-Wallis foram usados para avaliar os dados ecocardiográficos. O teste t pareado e o teste de Wilcoxon foram aplicados para avaliar a classe funcional.

A análise de regressão logística multivariada por *stepwise* foi realizada para os desfechos de interesse. As variáveis com um p-valor inferior a 0,20 na análise de regressão logística univariada foram selecionadas para a criação do modelo. Para a construção do modelo de regressão logística multivariada, a técnica *stepwise* foi usada pelo método de seleção *backward*. Para todas as análises estatísticas, incluindo a análise de regressão logística multivariada, um valor de p menor que 0,05 foi considerado estatisticamente significativo. A análise estatística e a construção dos gráficos foram conduzidas usando o programa Jamovi (versão 2.6.13) e R (versão 4.3.3), respectivamente.

## Resultados

### Características basais

Um total de 136 pacientes preencheram os critérios de inclusão e foram submetidos à CRVM no Instituto Nacional de Cardiologia, Rio de Janeiro, Brasil. As características basais são apresentadas na Tabela 1. A idade média dos pacientes foi 63,5±9,5 anos e 70,6% eram do sexo masculino, com FEVE média de 40,9 ± 8,6%. Hipertensão, dislipidemia e diabetes eram altamente prevalentes. Na admissão, a síndrome coronariana crônica (SCC) foi o principal diagnóstico, com mais de 65% dos pacientes em classe funcional III e IV. A avaliação da anatomia coronariana por angiografia mostrou uma elevada taxa de doença trivascular, lesão do tronco da coronária esquerda e da descendente anterior.

**Tabela 1:** – Características basais dos pacientes (n=136)

Características	n (%)
Idade, anos	63,5 ± 9,5
Sexo masculino	96 (70,6)
Hipertensão	124 (91,2)
Diabetes	80 (58,8)
Dislipidemia	102 (75)
Tabagismo (atual/anterior)	75 (55,1)
Sedentário	89 (66,9)
Doença cerebrovascular	36 (26,9)
Diagnóstico na admissão	
Síndrome coronariana crônica	122 (89,7)
Classe funcional CCS (escala de angina)	
I	7 (5,7)
II	35 (28,7)
III	56 (45,9)
IV	24 (19,7)
Insuficiência cardíaca	14 (10,3)
Classe funcional NYHA	
I	3 (21,4)
II	2 (14,3)
III	4 (28,6)
IV	5 (35,7)
TFGe, (mL/min/1,73m^2^)	75,9 (56,6; 91,0)
TFGe <60 (mL/min/1,73m^2^)	48 (35,3)
IMC (Kg/m^2^)	27,1 (24,8; 29,5)
FEVE, %	40,9 ± 8,6
Anatomia coronária	
LTCE ≥ 50%	45 (33,1)
DA ≥ 70%	131 (96,3)
DA proximal	92 (67,6)
Doença univascular	1 (0,7)
Doença bivascular	23 (16,9)
Doença trivascular	112 (82,4)
Euroscore II	1,89 (1,13; 2,86)

Dados apresentados em porcentagem, média ± desvio padrão ou mediana (intervalo interquartil); CCS: Canadian Cardiovascular Society; NYHA: New York Heart Association; TFGe: taxa de filtração glomerular estimada; IMC: índice de massa corporal; FEVE: fração de ejeção do ventrículo esquerdo; MC: índice de massa corporal; LTCE: lesão do tronco da coronária esquerda; DA: descendente anterior.

A CRVM com circulação extracorpórea foi realizada majoritariamente com uso de pontes arteriais (Tabela 2); cinco (3,7%) pacientes foram a óbito em 30 dias após a CRVM e 28 (20,9%) durante todo o período de acompanhamento. Uma alta taxa de infecção de ferida operatória foi observada durante o estudo.

**Tabela 2:** – Detalhes da cirurgia e desfechos

Variáveis	n = 136
Pontes arteriais *	129 (96,3)
Total de pontes	3,31 ± 0,99
Cirurgia com CEC (%)	124 (91,2)
Tempo de CEC (min)	98,7 ± 31,2
Mortalidade em 30 dias	5 (3,7)
IAM pós-operatório	5 (3,7)
Fibrilação atrial	23 (16,9)
AVC pós-operatório	4 (2,9)
Infecção da ferida operatória	25 (18,4)
Mediastinite	12 (8,8)

*Dados incompletos de dois pacientes; dados apresentados como média ± desvio padrão n (%); IAM: infarto agudo do miocárdio; CEC: circulação extracorpórea; AVC: acidente vascular cerebral.

### Tratamento clínico

Durante a internação para a intervenção, observamos que os pacientes apresentavam uma taxa elevada de uso de medicamentos com impacto cardiovascular, que foi mantido ao longo do acompanhamento (Tabela 3).

**Tabela 3:** – Adesão ao tratamento medicamentoso dos pacientes (n=136) com cardiomiopatia isquêmica submetidos à cirurgia de revascularização por bypass da artéria coronária entre 2013 e 2017

	Basal n = 135 (%)	Final do seguimento n = 97 (%)
Aspirina	132 (97,8)	91 (93,8)
Betabloqueador	129 (95,6)	87 (89,7)
IECA/BRA	100 (74,1)	81 (83,5)
Estatina	124 (91,2)	84 (87,5)

*Dados incompletos de um paciente; dados em n (%); IECA: Inibidor de Enzima Conversora de Angiotensina; BRA: Bloqueador de Receptor de Angiotensina

### Medidas ecocardiográficas após a revascularização

Na avaliação pré-operatória basal, os pacientes apresentavam extensa disfunção contrátil regional, com volumes aumentados do VE (Tabela 4). Em uma média de 3,6 meses após a CVRM, a FEVE aumentou de 40,9 ± 8,6% para 47,0 ± 12,6% (p<0,001) e a contratilidade regional também apresentou uma melhora significativa com uma redução no WMSI de 1,99 ± 0,36 para 1,80 ± 0,50 (p<0,001). Isso representa um aumento de 17,6% na FEVE e uma redução de 12,1% no WMSI. Tais resultados também foram acompanhados de uma importante diminuição nos valores medianos de volume e tamanho do VE. É importante salientar que a melhora nos parâmetros foi observada na análise comparativa entre o basal e o curto ou longo prazo, sem diferenças significativas entre os valores de curto e longo prazo.

**Tabela 4:** – Parâmetros ecocardiográficos de pacientes (n=136) com cardiomiopatia isquêmica submetidos à cirurgia de revascularização miocárdica entre 2013 e 2017

Variável	Pré-operatório	Curto prazo (3,6 meses)	Longo prazo (30,8 meses)	p
FEVE %	40,9 ± 8,6	47,0 ± 12,6	48,1 ± 15,0	p<0,001
WMSI	1,99 ± 0,36	1,80 ± 0,50	1,75 ± 0,49	p<0,001
VSF (mL)	97,3 (70,0:118,2)	78,6 (50,9:107,5)	78,0 (47,7:112,8)	p<0,001
VDF (mL)	166,6 (135,3:201,2)	147,4 (123,8:180,0)	147,4 (123,8:180,0)	p<0,001
DSF do VE (cm)	4,6 (4,0:5,0)	4,2 (3,5:4,8)	4,2 (3,4:4,9)	p<0,001
DDF do VE (cm)	5,8 (5,3:6,3)	5,5 (5,1:6,0)	5,5 (5,1:6,0)	p<0,001

Dados apresentados em média ± desvio padrão (intervalo interquartil); FEVE: fração de ejeção do ventrículo esquerdo; DDF: diâmetro diastólico final; DSF: diâmetro sistólico final; VSF: volume sistólico final; VDF: volume diastólico final; escore do índice de motilidade (WMSI, do inglês wall motion score index).

**Figura 1:**
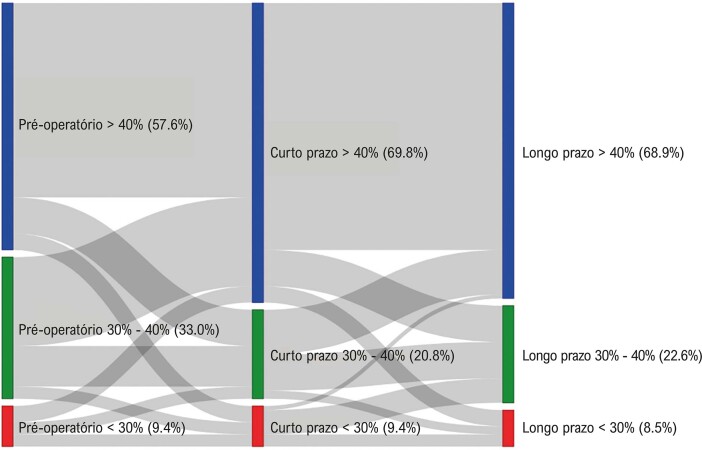
– Diagrama de Sankey apresentando a mudança na fração de ejeção do ventrículo esquerdo (%) do basal ao seguimento de curto prazo e seguimento de longo prazo.

Conforme demonstrado no diagrama de Sankey, que ilustra a dinâmica de mudança na FEVE durante o período de acompanhamento, a melhora é mais provável de ocorrer entre os pacientes com valores de fração de ejeção entre 30% e 40% no basal (Figura 1).

Observamos que 55,7% dos pacientes apresentaram um aumento na FEVE basal ≥ 10% e 58,1% apresentaram aumento no WMSI ≥10%. Na análise de regressão logística, a presença de doença cerebrovascular foi o único preditor de melhoria na FEVE≥10% (Tabela 5).

**Tabela 5:** – Predição de melhoria ≥10% na fração de ejeção do ventrículo esquerdo por regressão logística univariada

Variável	Beta estimado	SE	p	OR 95%
Doença trivascular	-0,378	0,52	0,47	0,68 (0,25-1,91)
FEVE<35%	0,846	0,53	0,11	2,33 (0,82-6,58)
Número de pontes inseridas ≥ 3	0,005	0,52	0,99	1,01 (0,36-2,79)
DA proximal ≥70%	-0,165	0,41	0,69	0,85 (0,38-1,91)
LTCE ≥ 50%	-0,176	0,41	0,67	0,84 (0,37-1,88)
Diabetes	0,165	0,37	0,65	1,18 (0,57-2,42)
Doença cerebrovascular	-1,532	0,48	0,001	0,22 (0,08-0,55)

FEVE: fração de ejeção do ventrículo esquerdo; OR: odds ratio; LTCE: lesão do tronco da coronária esquerda; DA: descendente anterior; SE: erro padrão.

### Avaliação clínica ao final do acompanhamento

Nesta coorte de pacientes, observamos uma melhora significativa na classe funcional com a CRVM (Figura Central). No pré-operatório, 65,4% dos pacientes eram muito sintomáticos e, ao final do acompanhamento, somente 10,3% permaneceram em classe funcional III ou IV (p<0,001).

**Figura Central:**
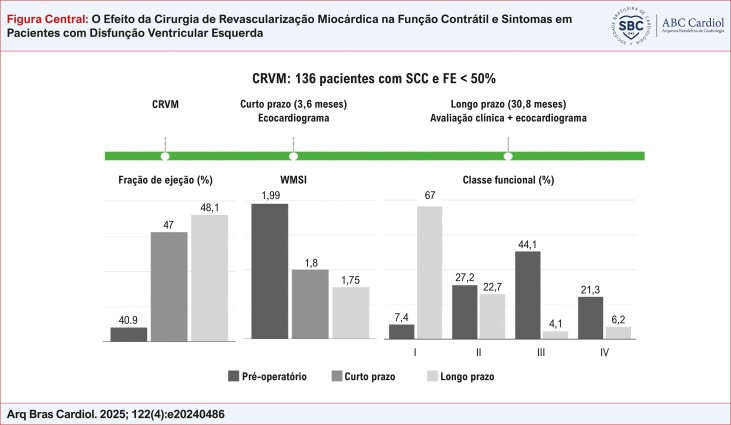
: O Efeito da Cirurgia de Revascularização Miocárdica na Função Contrátil e Sintomas em Pacientes com Disfunção Ventricular Esquerda

## Discussão

O presente estudo mostrou que, em pacientes com SCC com fração de ejeção reduzida, a CRVM associou-se com melhora na função do VE e regressão do volume e tamanho do VE. Além disso, a classe funcional melhorou no final do acompanhamento.

Estudos prévios demonstraram que 30-60% dos pacientes apresentam uma melhora na FEVE≥ 5% após a CRVM.^10-14^ Nosso estudo apresentou taxas ainda mais altas na recuperação da função do VE. Uma vez que as medicações exercem um importante papel no remodelamento do VE, é possível que o tratamento clínico otimizado durante o período de acompanhamento tenha influenciado esse resultado.^15,16^

O momento em que houve melhora da função do VE após a revascularização é um achado interessante. A melhoria na contratilidade foi sustentada no longo prazo, mas não houve incremento adicional após o ganho do curto prazo. Esses achados estão de acordo com publicações prévias, mostrando que uma resposta precoce ocorre no primeiro ano, mas sem melhorias adicionais ao longo do tempo.^12,17^

A coexistência de doença carotídea e doença arterial coronariana identifica um grupo de indivíduos com alta carga aterosclerótica e, portanto, com maior taxa de evento cardiovascular.^18,19^ Visto que pacientes com doença coronariana complexa e multivascular apresentam melhores resultados com a CRVM, isto que pode ser uma explicação para o papel da doença cerebrovascular como um preditor de melhora na função do VE.^20^

Após vários estudos observacionais, o STICH foi o primeiro ensaio randomizado a estabelecer o valor prognóstico da CRVM na insuficiência cardíaca isquêmica.^21^ Surpreendentemente, um subestudo identificou que a redução no volume do VE é mais provável de acontecer naqueles submetidos a CRVM do que nos que ficaram em tratamento conservador, mas o benefício prognóstico da revascularização não está relacionado a essa redução.^22^

## Conclusão

Pacientes com SCC e fração de ejeção reduzida submetidos à CRVM apresentaram uma melhora no curto prazo, tanto na função contrátil como no tamanho do VE, com uma resposta benéfica no longo prazo na classe funcional.

### Limitações

Este é um estudo retrospectivo e observacional, com desfechos cirúrgicos derivados de um único centro. Embora o estudo tenha envolvido um número razoável de pacientes, é necessário considerar que os exames de ecocardiografia não foram padronizados e foram realizados por diferentes operadores.
