# Supplementary material for: O Efeito da Cirurgia de Revascularização Miocárdica na Função Contrátil e Sintomas em Pacientes com Disfunção Ventricular Esquerda
Source: Arq Bras Cardiol. 2025 Apr 17;122(4):e20240486. [Article in Portuguese] doi: 10.36660/abc.20240486 (PMC12107766; doi:10.36660/abc.20240486)
Supplement: Translation

# Translation: The Effect of Coronary Artery Bypass Graft Surgery on Contractile Function and Symptoms in Patients with Left Ventricular Dysfunction

**DOI:** 10.36660/abc.20240486i

**Authors:** Fernando Bassan, Roberto Esporcatte, Marcelo Goulart Correia, Octavio Drummond Guina, Guilherme de Souza Weigert, Gracielle Christine do Nascimento Oliveira

**Affiliations:** 1 Brazil Instituto Nacional de Cardiologia, Rio de Janeiro, RJ – Brazil; 2 Brazil Universidade do Estado do Rio de Janeiro - Doenças do Tórax, Rio de Janeiro, RJ – Brazil

**Keywords:** Chronic Coronary Syndrome, Heart Failure, Left Ventricular Dysfunction, Myocardial Revascularization

## Abstract

**Background:**

The degree of left ventricular (LV) dysfunction is an independent risk factor for poor outcomes in patients with chronic coronary syndrome. Coronary artery bypass graft (CABG) is the standard care for the management of ischemic heart failure to improve symptoms and prognosis. However, the predictors of improvement are still uncertain.

**Objective:**

To assess the effect of myocardial revascularization on LV function and symptoms in patients with CCS and reduced left ventricular ejection fraction (LVEF), as well as to identify the improvement predictors.

**Methods:**

We retrospectively analyzed the data and clinical status of 136 consecutive patients with LVEF<50% that underwent CABG. During clinical follow-up echocardiographic LV function was reassessed at the short-term (3.6 months) and long-term (30.8 months), and compared to baseline.

**Results:**

Mean pre-operative LVEF was 40.9 ± 8.6% and wall motion score index (WMSI) was 1.99 ± 0.36, both improving at long-term to 48.1 ± 15.0% (p<0.001) and 1.75 ± 0.49 (p<0.001), respectively. We observed that 55.7% of the patients presented an improvement of LVEF≥10% and 58.1% in WMSI ≥10%. Univariate logistic regression analysis revealed that cerebrovascular disease was the only variable to be predictor of LVEF improvement. At the end of follow-up, we observed a reduction in the rate of patients in functional class III/IV when compared to baseline (65.4 vs. 10.3% - p<0.001).

**Conclusions:**

Patients with CCS and reduced LVEF undergoing CABG experienced improvement in both LV contractile function and size, with beneficial response in functional class.

**Central Illustration:** : The Effect of Coronary Artery Bypass Graft Surgery on Contractile Function and Symptoms in Patients with Left Ventricular Dysfunction

## Introduction

Ischemic cardiomyopathy remains the leading cause of death worldwide, and the degree of left ventricular (LV) dysfunction is one of the strongest prognostic marker. The etiology of heart failure is an important determinant, since patients with myocardial ischemia have lower survival as compared with non-ischemic patients.^1-4^

In heart failure, patients who experienced favorable changes in left ventricular ejection fraction (LVEF) had better prognosis.^5^ Revascularization with coronary artery bypass graft (CABG) is indicated to improve survival, but the mechanism of improvement is uncertain since neither the presence of ischemia nor viability predicted the benefit.^6,7^

In this study, we aimed to analyze the effect of CABG on LV function in patients with ischemic cardiomyopathy and reduced ejection fraction.

## Methods

### Study population

This was a retrospective, observational, cohort study of consecutive, non-selected patients with ischemic cardiomyopathy that underwent CABG surgery from 2013 to 2017 in our institution. Eligible patients had (1) LVEF <50% assessed by echocardiography in the preoperative period; (2) no need for LV reconstruction or associated cardiac valve intervention. Since acute ischemic events could be a strong bias for LV function recovery, we also excluded acute coronary syndrome patients with any event two months prior to surgery.

### Patient management

CABG indication has been decided at discretion of a multidisciplinary team composed of clinical cardiologists and cardiac surgeons. Euroscore II was used to assess the pre-operative risk. Post-operative stroke was defined as a new or worsening focal neurological event that persisted for >24h during the hospitalization period. Post-operative myocardial infarction was defined according to the third universal definition of myocardial infarction.^8^ Cerebrovascular disease was defined as carotid stenosis ≥70% unilateral or ≥50% bilateral. After hospitalization, patients were followed regularly in outpatient settings. We performed a final evaluation of all patients at the end of the follow-up to check clinical status for analysis in this study.

### Echocardiographic analysis

Patients were submitted to resting transthoracic echocardiogram (TTE) before the CABG (baseline), in the first six months after surgery (short-term) and at the end of the follow-up (long-term). TTE parameters were measured following standard recommendations.^9^ LV wall motion score index (WMSI) was measured using a 17-segment model. The following numerical score was assigned to each wall segment according to its contractile function as assessed visually: 1=normal; 2=hypokinetic; 3=akinetic; 4=dyskinetic; all defined by visual assessment. The WMSI was calculated as the sum of all scores divided by the total number of segments.

### Statistical analysis

Categorical data were described using frequency and percentage. Continuous variables were assessed for normality using the Shapiro-Wilk test and described as mean and standard deviation or median with interquartile range. Chi-square and Fisher´s exact tests were used to determine statistical associations between variables of interest. Repeated measures ANOVA and Kruskal-Wallis tests were used for echocardiographic data assessment. Paired t-test and Wilcoxon test were applied for functional class evaluation.

Multivariate stepwise logistic regression analysis was carried out for the outcomes of interest. Those variables with a p-value below 0.20 in the univariate logistic regression analysis were selected for the creation of the model. For the construction of the multivariate logistic regression model, the stepwise technique was used through the backward variable selection method. For all the statistical analyses, including the multivariate logistic regression analysis, a p-value less than 0.05 was considered to be statistically significant. The statistical analysis and construction of the graphs were performed using the software Jamovi (version 2.6.13) and R (version 4.3.3), respectively.

## Results

### Baseline characteristics

One hundred and thirty six patients met the inclusion criteria and underwent CABG at the National Institute of Cardiology, Rio de Janeiro, Brazil. Baseline characteristics are shown in Table 1. Mean age of patients was 63.5±9.5 years and 70.6% were male, with a mean LVEF of 40.9 ± 8.6%. Hypertension, dyslipidemia and diabetes were highly prevalent. On admission, chronic coronary syndrome was the main diagnosis with over 65% of the patients highly symptomatic, with functional class III and IV. Coronary anatomy assessment by coronariography has shown a high rate of three-vessel disease, and involvement of left main and left anterior descending artery.

**Table 1:** – Baseline characteristics of the patients (n=136)

Characteristics	n (%)
Age, years	63.5 ± 9.5
Male gender	96 (70.6)
Hypertension	124 (91.2)
Diabetes	80 (58.8)
Dyslipidemia	102 (75)
Smoking (current/former)	75 (55.1)
Sedentary	89 (66.9)
Cerebrovascular disease	36 (26.9)
Admission diagnosis	
Chronic coronary syndrome	122 (89.7)
CCS (angina scale) functional class	
I	7 (5.7)
II	35 (28.7)
III	56 (45.9)
IV	24 (19.7)
Heart failure	14 (10.3)
NYHA functional class	
I	3 (21.4)
II	2 (14.3)
III	4 (28.6)
IV	5 (35.7)
eGFR, (ml/min/1.73m^2^)	75.9 (56.6; 91.0)
eGFR <60 (ml/min/1.73m^2^)	48 (35.3)
BMI (kg/m^2^)	27.1 (24.8; 29.5)
LVEF, %	40.9 ± 8.6
Coronary anatomy	
LMCA ≥ 50%	45 (33.1)
LAD ≥ 70%	131 (96.3)
Proximal LAD	92 (67.6)
One-vessel disease	1 (0.7)
Two-vessel disease	23 (16.9)
Three-vessel disease	112 (82.4)
Euroscore II	1.89 (1.13; 2.86)

Data are presented as percentage, mean value ± standard deviation or median (interquartile range); CCS: Canadian cardiovascular society; NYHA: New York Heart Association; eGFR: estimated glomerular filtration rate; BMI: body mass index; LVEF: left ventricle ejection fraction; LMCA: left main; LAD: left anterior descending.

On-pump surgery was mostly performed with arterial conduits (Table 2); five (3.7%) patients died within 30 days after CABG and 28 (20.9%) during the entire follow-up. A high rate of surgical infection was observed during the study.

**Table 2:** – Operative details and outcomes

Variables	n = 136
Arterial conduits*	129 (96.3)
Total grafts	3.31 ± 0.99
On-pump surgery (%)	124 (91.2)
CPB time (min)	98.7 ± 31.2
30-day mortality	5 (3.7)
Postoperative MI	5 (3.7)
Atrial fibrillation	23 (16.9)
Postoperative stroke	4 (2.9)
Wound infection	25 (18.4)
Mediastinitis	12 (8.8)

*2 patients with missing data. Data are presented as mean value ± standard deviation or n (%). CPB: cardiopulmonary bypass; MI: myocardial infarction.

### Medical therapy

At the index hospitalization for the intervention, we observed that patients had a high rate use of medications with cardiovascular impact that was maintained throughout the follow-up (table 3).

**Table 3:** – Adherence to drug treatment of patients (n=136) with ischemic cardiomyopathy who underwent coronary artery bypass graft surgery between 2013 and 2017

	Baseline n = 135 (%)	End of follow-up n = 97 (%)
Aspirin	132 (97.8)	91 (93.8)
Beta-blocker	129 (95.6)	87 (89.7)
ACEi/ARB	100 (74.1)	81 (83.5)
Statin	124 (91.2)	84 (87.5)

*1 patient with missing data. Data are n (%). ACEi: angiotensin-converting enzyme inhibitor; ARB: angiotensin receptor blocker.

### Echocardiographic measures after revascularization

At baseline, patients presented extensive regional contractile dysfunction with enlarged LV volumes (Table 4). At a mean of 3.6 months after CABG, LVEF increased from 40.9 ± 8.6% to 47.0 ± 12.6% (p<0.001) and regional contractility also presented significant improvement with a reduction in the WMSI from 1.99 ± 0.36 to 1.80 ± 0.50 (p<0.001). This represents an increase of 17.6% in LVEF and a reduction of 12.1% in the WMSI. Those results were also accompanied by a major decrease in median LV volume and size. It is important to notice that those improvements are restricted to the analysis between preoperative and short-term or long-term measurements, with non-significant differences between short-term and long-term values.

**Table 4:** – Echocardiographic parameters of patients (n=136) with ischemic cardiomyopathy who underwent coronary artery bypass graft surgery between 2013 and 2017

Variable	Preoperative	Short-term (3.6 months)	Long-term (30.8 months)		p
LVEF %	40.9 ± 8.6	47.0 ± 12.6	48.1 ± 15.0	p<0.001	
WMSI	1.99 ± 0.36	1.80 ± 0.50	1.75 ± 0.49	p<0.001	
ESV (ml)	97.3 (70.0:118.2)	78.6 (50.9:107.5)	78.0 (47.7:112.8)	p<0.001	
EDV (ml)	166.6 (135.3:201.2)	147.4 (123.8:180.0)	147.4 (123.8:180.0)	p<0.001	
LVESD (cm)	4.6 (4.0:5.0)	4.2 (3.5:4.8)	4.2 (3.4:4.9)	p<0.001	
LVEDD (cm)	5.8 (5.3:6.3)	5.5 (5.1:6.0)	5.5 (5.1:6.0)	p<0.001	

Data are presented as mean value ± standard deviation or median (interquartile range). LVEF: left ventricle ejection fraction; WMSI: wall motion score index; ESV: end-systolic volume; EDV: end-diastolic volume; LVESD: left ventricular end-systolic diameter; LVEDD: left ventricular end-diastolic diameter.

As shown by the Sankey diagrams delineating the dynamic change of LVEF over the entire follow-up, the improvement is more likely to occur among patients with ejection fraction values between 30% and 40% at baseline (Figure 1).

**Figure 1:** – Sankey diagrams depicting the change in LVEF (%) from baseline to short and long-term follow-up.

We found that 55.7% of the patients presented ≥10% increase of baseline LVEF and 58.1% presented an improvement ≥10% of the WMSI.

The logistic regression analysis revealed that the presence of cerebrovascular disease was the only predictor of improvement ≥ 10% in LVEF (Table 5).

**Table 5:** – Univariate logistic regression prediction for improvement of LVEF≥10%

Variable	Beta estimate	SE	p	OR 95%
Three vessel disease	-0.378	0.52	0.47	0.68(0.25-1.91)
LVEF<35%	0.846	0.53	0.11	2.33(0.82-6.58)
Number of grafts inserted ≥ 3	0.005	0.52	0.99	1.01(0.36-2.79)
Proximal LAD ≥70%	-0.165	0.41	0.69	0.85(0.38-1.91)
LM ≥ 50%	-0.176	0.41	0.67	0.84(0.37-1.88)
Diabetes	0.165	0.37	0.65	1.18(0.57-2.42)
Cerebrovascular disease	-1.532	0.48	0.001	0.22 (0.08-0.55)

LVEF: left ventricle ejection fraction; LAD: left anterior descendent; LM: left main; OR: odds ratio; SE: standard error.

### Clinical assessment at the end of the follow-up

In this cohort of patients, we observed a significant improvement in symptoms with CABG (Central Figure). Preoperatively, 65.4% of patients were highly symptomatic, while at the end of follow-up, only 10.3% remained in functional class III or IV (p<0.001).

## Discussion

The present study has shown that in patients with chronic coronary syndrome with reduced ejection fraction, CABG was associated with improvement in LV function and regression of LV volume and size. In addition, functional class improved at the end of the follow-up.

Prior studies have demonstrated that 30-60% of the patients present an improvement ≥ 5% in LVEF after CABG.^10-14^ Our study presented even higher rates in the LV function recovery. Since medication plays an important role on LV remodeling, it is possible that optimal medical treatment during the follow-up could have influenced this result.^15,16^

The timing of the LV function improvement after revascularization is an interesting finding. Contractility improvement was sustained in the long-term, but there were no additional increments after the short-term follow-up. These findings are in harmony with previous publication, showing that an early response occurs in the first year, but without additional improvements over time.^12,17^

The coexistence of carotid and coronary artery disease identified a group of individuals with high atherosclerotic burden and, therefore, with an elevated level of cardiovascular event.^18,19^ Since multivessel and complex coronary artery disease patients have better results with CABG, this can be an explanation for the role of cerebrovascular disease as a predictor of LV function improvement.^20^

After several observation studies, STICH was the first randomized trial to establish the prognosis value of CABG in ischemic heart failure.^21^ Surprisingly, a sub-study found that the reduction in LV volume is more likely to occur in CABG than in the conservative arm, but the revascularization prognosis benefit is not linked to this reduction.^22^

## Conclusion

Patients with chronic coronary syndrome and reduced ejection fraction undergoing CABG experienced short-term improvement in both LV contractile function and size, with beneficial long-term response in their functional class.

### Limitations

This is a retrospective and observational study, with operative outcomes of a single center. Even though the study involved a reasonable number of patients, it is necessary to consider that the echocardiograms were not standardized and were performed by different operators.
